# The Occurrence of *Photorhabdus*-Like Toxin Complexes in *Bacillus thuringiensis*

**DOI:** 10.1371/journal.pone.0018122

**Published:** 2011-03-25

**Authors:** Michael B. Blackburn, Phyllis A. W. Martin, Daniel Kuhar, Robert R. Farrar, Dawn E. Gundersen-Rindal

**Affiliations:** Invasive Insect Biocontrol and Behavior Laboratory, Agricultural Research Service, United States Department of Agriculture, Henry A. Wallace Beltsville Agricultural Research Center, Beltsville, Maryland, United States of America; The University of Hong Kong, Hong Kong

## Abstract

Recently, genomic sequencing of a *Bacillus thuringiensis* (*Bt*) isolate from our collection revealed the presence of an apparent operon encoding an insecticidal toxin complex (Tca) similar to that first described from the entomopathogen *Photorhabdus luminescens*. To determine whether these genes are widespread among *Bt* strains, we screened isolates from the collection for the presence of *tccC*, one of the genes needed for the expression of fully functional toxin complexes. Among 81 isolates chosen to represent commonly encountered biochemical phenotypes, 17 were found to possess a *tccC*. Phylogenetic analysis of the 81 isolates by multilocus sequence typing revealed that all the isolates possessing a *tccC* gene were restricted to two sequence types related to *Bt* varieties *morrisoni*, *tenebrionis*, *israelensis* and *toumanoffi*. Sequencing of the ∼17 kb *tca* operon from two isolates representing each of the two sequence types revealed >99% sequence identity. Optical mapping of DNA from *Bt* isolates representing each of the sequence types revealed nearly identical plasmids of ca. 333 and 338 kbp, respectively. Selected isolates were found to be toxic to gypsy moth larvae, but were not as effective as a commercial strain of *Bt kurstaki*. Some isolates were found to inhibit growth of Colorado potato beetle. Custom Taqman® relative quantitative real-time PCR assays for Tc-encoding *Bt* revealed both *tcaA* and *tcaB* genes were expressed within infected gypsy moth larvae.

## Introduction

**Figure 1:**
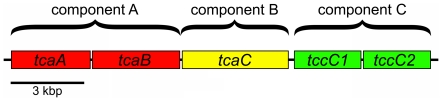
Organization of the *tca* operon as found in *Bt* isolate IBL 200.

In 1998 Bowen et al. [1] described a novel class of insecticidal toxin complexes, or Tcs, from the nematode symbiont *Photorhabdus luminescens*. In the years since, genes for related toxins have been revealed in a number of gram-negative bacteria such as *Xenorhabdus spp.*, *Yersinia spp.*, and *Serratia spp.* [2]–[4]. More recently, genomic sequencing of *Bacillus thuringiensis* (*Bt*) isolate IBL 200 from the Invasive Insect Biocontrol and Behavior Laboratory (IIBBL; Beltsville, MD, USA) collection revealed the presence of an apparent toxin-encoding operon similar to *tca* of *P. luminescens* W14 [1], and reports of similar *tca* genes in a *Paenibacillus sp*. and in *Bt* have appeared in the patent literature [5], [6]. Using the nomenclature of Bowen et al. [1], IBL 200 possesses an apparent five gene operon consisting of, sequentially; *tcaA*, *tcaB*, *tcaC*, and two consecutive *tccC*s (GenBank accession number NZ_ACNK01000119). Based on studies of how the subunit proteins encoded by these genes interact, the operon appears to encode a fully active toxin complex [7]. Adopting the “ABC” toxin component scheme suggested by ffrench-Constant et al. [8], the IBL 200 operon is complete in that components A (*tcaA* and *tcaB*), B (*tcaC*), and C (*tccC*) are present (Figure 1).

Intrigued by these revelations of *tca* genes occurring in Gram-positive entomopathogens, we wished to determine how prevalent these genes might be in our *Bt* collection, and gain insight into their phylogenetic distribution. Eighty-one *Bt* isolates selected from the IIBBL collection for their phenotypic diversity were phylogenetically characterized by multilocus sequence typing and screened for the presence of *tc* genes. We also examined the expression of *tc* genes by a tca-bearing Bt during infection of gypsy moth larvae by means of real-time qPCR.

## Results

### Detection of tccC and sequencing

Among 81 *Bt* isolates chosen from the IIBBL collection to represent prevalent biochemical phenotypes (Table S1) [9], a total of 17 isolates possessed a *tccC* gene based on PCR screening using degenerate primers for *tccC*. The primers employed were also successful in producing *tccC* amplicons from *P. luminescens* W14 [1], and from the *tca*-positive *Bt* isolates NRRL-B-30758, NRRL-B-30759 and NRRL-B-30760 described elsewhere [6]. Sequencing revealed that the 347 bp *tccC* amplicons from all 17 isolates were identical. Sequences of the *tca* operon were determined for IBL 90, IBL 122, IBL 500 and IBL 888 using non-degenerate primers based on the sequence of the operon from IBL 200. For each of these four strains, the sequence of the 17-kb operon was found to be essentially identical to that of IBL 200.

### Phylogenetic relationships among tc-positive isolates

Utilizing the multilocus sequence typing scheme of Priest et al. [10], phylogenetic analyses using parsimony revealed that the 17 *tc*-positive *Bt* isolates were restricted to two sequence types. Seven *tc*-positive isolates (IBL 122, IBL 273, IBL 275, IBL 500, IBL 3090 and IBL 3579) belonged to ST 23 and were characterized phenotypically by their production of bipyramidal crystals, toxicity to lepidopteran species, the absence of lecithinase and urease production, and the ability to produce acid from sucrose (only IBL 3090 lacked this trait). The only representative of ST 23 from our sample that appeared to lack *tccC* (IBL 1410) was a *Bt tenebrionis* strain derived from a preparation of Novodor® (Mycogen, San Diego, CA, USA). In contrast to the *tc*-positive members of this group, the *tenebrionis* isolate produced rectangular flake-like crystals, and was not toxic to lepidopteran species. Ten *tc*-positive *Bt* isolates (IBL 26, IBL 54, IBL 61, IBL 90, IBL 110, IBL 200, IBL 888, IBL 950, IBL 1115 and IBL 1140) belonged to ST 240, which until this report, had been represented by a single isolate in the PubMLST *Bacillus cereus* database [11]. Unlike ST 23, the ST 240 isolates produced both lecithinase (only IBL 950 lacked this trait) and urease, and did not produce acid from sucrose. Like the *tc*-positive members of ST 23, isolates within ST 240 produced bipyramidal crystals and were toxic to lepidopteran species. All isolates that we identified as ST 240 were *tc*-positive. Among three *Bt* isolates previously identified as *tc*-positive [6], NRRL-B-30759 and NRRL-B-30760 possessed *tca* operons essentially identical to those found in our collection, and were identified as ST 23. NRRL-B-30758, displaying a divergent nucleotide sequence across the entire *tca* operon, was found to belong to ST 8. The 64 tc-negative isolates from the IIBBL collection were distributed among ST 8 (26 isolates), ST 171 (12 isolates), ST 16 (7 isolates), ST 241 (4 isolates), ST 22 (2 isolates), ST 197 (2 isolates), ST 10 (1 isolate), ST 23 (1 isolate), ST 26 (1 isolate), ST 239 (1 isolate), and 5 novel STs (7 isolates).

### Optical mapping of plasmids from ST 23 and ST 240

**Figure 2:**
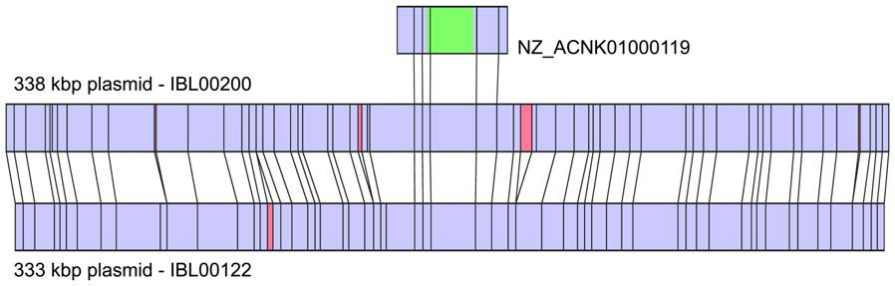
Optical maps of the 338 kbp and 333 kbp plasmids from IBL 200 and IBL 122, respectively, as cleaved by XbaI. Highly similar areas are shaded in blue, while dissimilar regions are red. The smaller map above the plasmids represents the predicted XbaI cleavage of the 42 kbp genomic sequence from IBL 200 bearing the *tca* operon (location shaded in green).

Optical mapping of IBL 200 (ST 240) genomic DNA revealed two large plasmids of ca. 338 kb and 521 kb. When partially assembled genomic sequences of IBL 200 were aligned with these maps, a number of contigs appeared to be associated with the plasmids. One of these, a 42 kb fragment (GenBank accession number NZ_ACNK01000119) containing the entire *tca* operon, had predicted Xba1 sites consistent with those found on a region of the 338 kb plasmid. Other contigs with predicted Xba1 sites aligning with those of the 338 kb plasmid (NZ_ACNK01000108, NZ_ACNK01000099, NZ_ACNK01000232, NZ_ACNK01000120 and NZ_ACNK01000123) bore multiple copies of genes encoding Cry1Ae, Cry1Bc and several vegetative insecticidal proteins. Optical mapping of IBL 122, representing ST 23, revealed plasmids of 333 kb and 284 kb. The Xba1 restriction pattern of the 333 kb plasmid from IBL 122 was found to be highly similar to that of the 338 kb plasmid of IBL 200 (Figure 2). No detectable similarities were found between the 521 kb plasmid of IBL 200 and the 284 kb plasmid of IBL 122. Optical mapping of IBL 1410, a *Bt tenebrionis* isolate lacking detectable *tc* genes, possessed a 334 kb plasmid with no similarity to the *tca* bearing plasmids of IBL 200 or IBL 122 (data not shown).

### Insect toxicity

Mortality of gypsy moth larvae was significantly affected by isolate (*F*_6, 18_ = 16.75, *P* = 0.0001). Although all 17 isolates possessing *tccC* were found to produce bipyramidal crystals and had previously been found toxic to lepidopteran larvae, sporulated cultures of selected *tc*-positive isolates were found to be substantially less active against gypsy moth larvae than was IBL 455, which was isolated from a 1980 preparation of Dipel® (Abbot Laboratories, Chicago, IL, USA). The relative activities of the *tc*-positive strains, on a culture-equivalent basis, were not statistically different from each other (Table 1). Control mortality was less than that caused by any *Bt* treatment.

**Table 1:** Mortality of gypsy moth larvae fed Bt isolates with *tca* genes compared with a DiPel derived strain (IBL 455).

Isolate	tccC	Sequence type	% Mortality
IBL 455	−	8	82.3^A^
IBL 90	+	240	24.0^B^
IBL 26	+	240	24.0^B^
IBL 3090	+	23	22.9^B^
IBL 747	+	23	19.8^B^
IBL 122	+	23	17.7^B^
Control	N/A	N/A	3.1^C^

Percentages followed by different letters are significantly different (*P*>0.05) by LSD.

Weights of Colorado potato beetle larvae were significantly affected by *Bt* isolate (*F*_7, 114_ = 13.59, *P* = 0.0001) (Table 2). Some *tc*-positive isolates reduced larval weight relative to the *tc*-negative isolate (IBL 441), while others did not.

**Table 2:** Weights of Colorado potato beetle larvae fed whole cultures of *tc*-positive and *tc*-negative *B. thuringiensis* isolates.

Isolate	*tccC*	Sequence type	Mean weight in mg ± SE
Control	N/A	N/A	12.10±0.849^A^
IBL 61	+	240	11.73±1.157^AB^
IBL 441	−	8	10.52±0.742^BC^
IBL 1140	+	240	9.94±0.384^BCD^
IBL 950	+	240	9.38±0.769^CD^
IBL 747	+	23	7.86±0.863^D^
IBL 3090	+	23	5.30±0.345^E^
IBL 122	+	23	4.98±0.420^E^

Means followed by different letters are significantly different (*P*>0.05) by LSD.

### Expression of tca during pathogenesis

**Figure 3:**
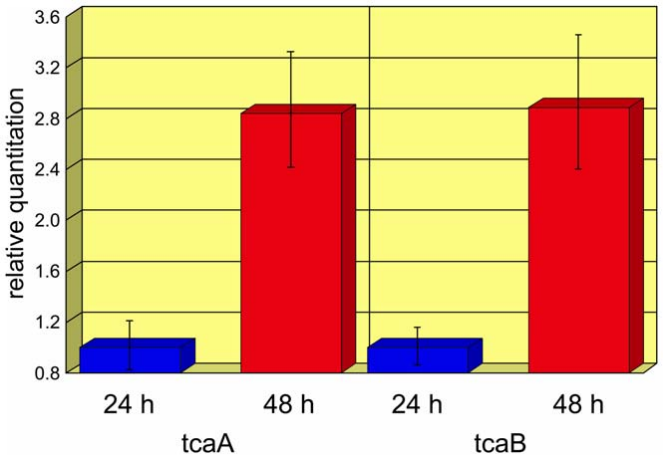
Relative expression of *tcaA* and *tcaB* by IBL 90 in infected gypsy moth larvae at 24 h and 48 h.

Expression of *Bt*-encoded *cry1*, *tcaA* and *tcaB* toxin genes was analyzed within *L. dispar* larvae infected separately with *Bt*s IBL 455 and IBL 90 at time points 24 h and 48 h post infection (Figure 3). In vivo *cry1* expression by both isolates was detected at 24 h and 48 h post infection, while *tcaA* and *tcaB* expression was only detected by IBL 90 as expected. Relative to levels observed at 24 h, the expression of *tcaA*, *tcaB*, *cry1*, and *glpf* (glycerol uptake facilitator protein) by IBL 90 at 48 h each increased by a factor of approximately 3.

## Discussion

**Figure 4:**
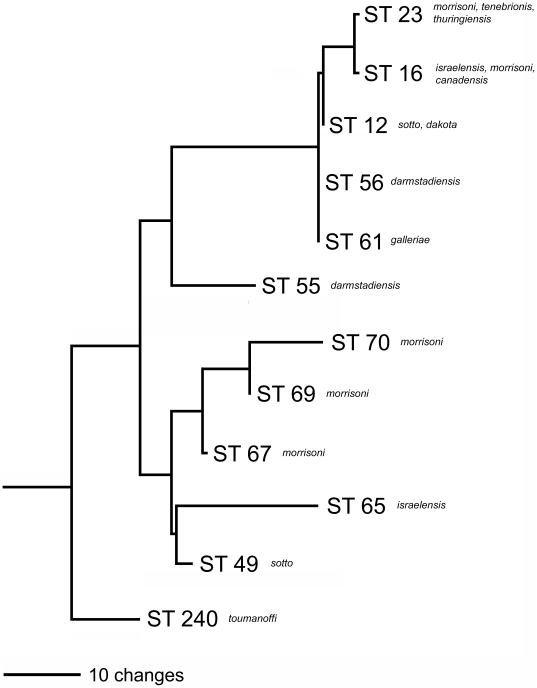
Phylogenetic relationship of ST 23 and ST 240 to other STs within the Sotto cluster of *Bacillus cereus* group bacteria. *Bt* serotypes occurring within a given ST are indicated.

Among 81 phenotypically diverse *Bt* isolates from the IIBBL collection, we identified 17 possessing *tccC* genes. The *tc*-positive isolates were cultured from 13 soil samples originating from the northern United States, Sweden, Norway and Iceland. These isolates were found to belong exclusively to two sequence types, ST 23 and ST 240, within the previously described “Sotto” group [10] which includes isolates of *Bt* varieties *sotto*, *israelensis*, *morrisoni* and *tenebrionis* (Figure 4). Interestingly, one soil sample from a Long Island (NY) sand dune yielded *tc*-positive isolates belonging to both ST 23 (IBL 3090) and ST 240 (IBL 110). Nearly all isolates (17 of 18) belonging to ST 23 and ST 240 were *tc*-positive. Sequence typing of three additional *tca*-containing *Bt* strains described in the patent literature [6] revealed that NRRL-B-30759 and NRRL-B-30760 belong to ST 23, while NRRL-B-30758 belongs to ST 8. Although our study included 26 ST 8 isolates from our collection, we did not detect any *tc*-positives among them. Thus it appears that the frequency of *tc* genes among ST 23 and ST 240 isolates is very high, but considerably lower within ST 8.

The IIBBL collection is composed almost exclusively of *Bts* from soil, generally isolated by acetate selection [12], with the only requirements for inclusion in the collection being a *B. cereus* biochemical phenotype and the production of a parasporal crystal [9]. Assuming that the collection represents a relatively unbiased cross-section of *Bt* as it exists in soil, our results suggest that the frequency of *tc*-positive *Bt* in the environment is surprisingly high. Isolates belonging to ST 23 and ST 240 were abundant in our sample, and were nearly always *tc*-positive.

With 10 isolates in the PubMLST database [11], ST 23 appears to be commonly encountered by other researchers as well; only ST 8 and ST 10 (primarily varieties *kurstaki* and *thuringiensis*, respectively) are better represented. Among the ST 23 in the database are five isolates of variety *morrisoni*, a single *tenebrionis*, and four isolates recovered in the vegetative state from the phylloplane of clover [13]. In contrast, the 10 isolates of ST 240 in our sample are surprising considering that this sequence type is represented in the database by a single isolate of *Bt toumanoffi* exhibiting weak mosquitocidal activity against *Culex spp.* (Terrance Leighton and Katie Wheeler; unpublished data). While some of this discrepancy can be explained by our inclusion of a larger number of isolates having the same biochemical phenotype as IBL 200, our sample still seems unusually rich in this apparently rare sequence type. The high incidence of ST 240 does not appear due to a sampling anomaly; the 10 isolates identified in this study originated from 9 widely separated samples. We can only speculate that *Bt* collections may generally be biased towards strains with more impressive insecticidal activity than ST 240 isolates appear to have. While we are comfortable with suggesting that the occurrence of *tc* genes among *Bt* in soil is fairly common, there are many sequence types not included in our sample; obviously, an accurate estimate of *tc* distribution among sequence types cannot be made here, and will require substantially more effort.

**Figure 5:**
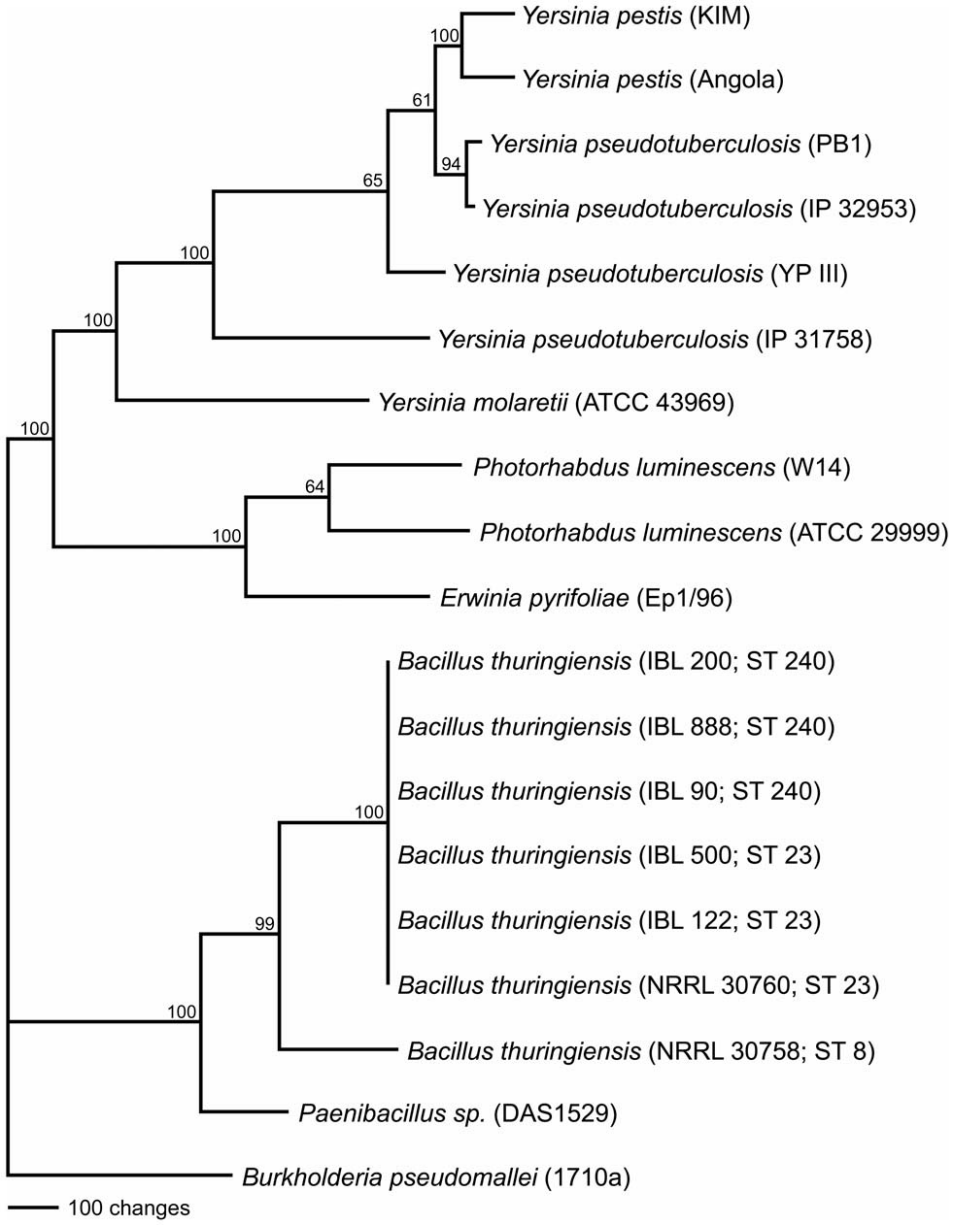
Phylogenetic tree based on TcaC amino acid sequences inferred from *tca* operons occurring in different bacteria. Only TcaCs encoded within *tca* operons were included in the analysis (TcaCs encoded in tcd-like operons were not included).

The G+C content of the *tca* genes is quite similar to the rest of the genome in *Bt*, ca. 35%, suggesting that the genes have been associated with *Bt* for some time. Phylogenetic analysis of amino acid sequences inferred by *tcaC* genes found in bacteria with true *tca* operons (possessing *tcaA* and *tcaB*) suggest that *Bt* did not acquire *tca* from either *Photorhabdus spp.* or *Yersinia spp.* in recent times, but from a more distant common ancestor (Figure 5). The near identity of *tca* sequences found in ST 23 and ST 240 would seem to indicate recent horizontal transfer of the operon between the two, as there is less sequence variation among the *tc* genes from the two STs than there is among the housekeeping genes employed in the MLST scheme. Optical mapping of genomic DNA from representatives of ST 23 and ST 240 leave little doubt that the horizontal transfer of *tca* occurred via plasmid exchange. The homogeneity of tc sequences we encountered among ST 23 and ST 240, and plasmid similarities between the two types suggest that the plasmid may move relatively freely between members of ST 23 and ST 240, and that newer and more adaptive versions of the plasmid periodically displace older less adaptive ones. The apparent similarities in geographic distribution of ST 23 and ST 240, and the isolation of *tc*-positive isolates of both STs from a single soil sample, as noted in the preceding discussion, would seem to support such a possibility.

Based on our limited results, *tc*-bearing *Bts* do not appear to be comparatively superior candidates for use as control agents. However, nothing is known about how *tca* is deployed by the bacterium against insects, or against which insects. While our preliminary experiments detect expression of both *tcaA* and *tcaB* by isolate IBL 90 during infection of gypsy moth larvae, we have not yet established expression profiles across the entire lifecycle of the bacterium. It seems unlikely that Tca would be expressed in the same fashion as the δ-endotoxins due to stability issues. The crystalline endotoxins presumably persist in the environment alongside the dormant spores, and can exert their effect on the midgut before the *Bt* spores even germinate. It seems unlikely that Tca would persist for very long after *Bt* has sporulated, and that whatever benefit the bacterium derives from the toxin is more temporally correlated with expression than is the case for the endotoxins.

Despite the fact that Tca is quite toxic to Colorado potato beetle [14], the *tc*-positive isolates that we tested against the beetle were only modestly active. Interestingly, isolates of ST 23, which also includes *Bt tenebrionis*, were consistently better at inhibiting growth of beetle larvae than isolates of ST 240. However, the only representative of ST 23 we found lacking a *tccC* was IBL 1410, a true *tenebrionis* isolate; optical mapping of this isolate revealed that its single large plasmid was unrelated to either of those carried by *tc*-positive members of ST 23.

Given the distribution of Tcs among other insect-associated bacteria, it is probably not surprising to discover versions of these toxins in *Bt*. The Tcs have probably escaped detection in *Bt* thus far due to their seemingly subtle contribution to pathogenesis. With the advent of genomic sequencing, it seems likely that more such revelations will occur in the near future.

## Materials and Methods

### Selection of isolates

Eighty-one diverse Bt isolates representing commonly encountered phenotypic profiles were chosen for phylogenetic analysis from the IIBBL *Bt* collection based on substrate utilization tests. Briefly, six traits that occurred in 20–86% of the 3429 isolates in the collection were used to form a classification system. These traits included hydrolysis of starch (T, 85.8% of isolates), production of phospholipase C or lecithinase (L, 79.7% of isolates), production of urease (U, 20.5% of isolates), acid production from sucrose (S, 34.0% of isolates), acid production from salicin (A, 37.4% of isolates), and hydrolysis of esculin (E, 32.3% of isolates). Based on the frequencies of trait combinations in the entire collection, representative isolates of the more common phenotypes were selected for study (Table S1). An additional selection of isolates displaying the same phenotype as IBL 200 (TLU) was included. The methods used to perform the substrate utilization tests, and the relative frequencies of phenotypes in the collection are discussed fully in Martin et al. [9].

### Tc screening and phylogenetic analysis

Bacteria were grown in L-medium [15] for 8 h at 25°C on an orbital shaker at 250 rpm. DNAs were isolated using the Quantum Prep miniprep kit (BioRad, Hercules, CA, USA) as specified by the manufacturer for use as template in polymerase chain reaction (PCR). 

Degenerate PCR primers for *tccC* (*tccC*F1: CTCACCATRCGATATAAATT and *tccC*R1: CAAGTMMGGGTATTACATTGG) were developed based on the sequences of these genes in IBL 200 and *P. luminescens* W14 [1], [16]. Since all amplicons obtained from IIBBL collection isolates with these primers had sequences identical to that of IBL 200, subsequent primer sets designed to amplify the entire *tca* operon were non-degenerate and based upon the IBL 200 sequence.

Amplification, sequencing, and sequence type analysis, and was performed using the multilocus sequence typing (MLST) scheme of Priest et al. [10]. Sequences for the multiple loci were amplified for each isolate using primers for the *glpf*, *gmk*, *pta*, *tpi*, *ilvD*, *pur*, and *pycA* loci as designed and described in that earlier study, with the exception of a new *pta* forward primer (*pta*F1 5′- GCGTTTAGCAAAAGAAGAGTTAGTA -3′) designed in this study. For PCR, thirty-five cycles were conducted in a model 9700 thermocycler (Applied Biosystems, Foster City, CA, USA) using 30 sec denaturation at 94°C, 1.5-min annealing at 55°C, and 2-min primer extension (10-min in final cycle) at 72°C. Each gene amplicon was sequenced directly. Products were separated on 1.5% NuSieve agarose gel (FMC, Rockland, ME) in modified-1× TAE (0.04 M Tris-acetate and 0.1 mM EDTA), and excised for sequencing using ABI BigDye V1.1 (Applied Biosystems, Foster City, CA, USA), using the amplification primers. Cycle sequencing conditions were 35 cycles at 96°C, 10 sec; 50°C, 5 sec; 60°C for 4 min. Automatic sequencing was carried out on an ABI Prism Model 3130×l (Applied Biosystems, Foster City, CA, USA). Sequences were edited and assembled using the SeqMan component of DNASTAR (DNASTAR, Inc. Madison, WI, USA). Sequence types were determined by BLAST searches [17] of the PubMLST database for the *Bacillus cereus* group [11]. Phylogenetic placement of *tc*-positive strains among STs having three or more isolates in the database (Table S2) was accomplished using parsimony by heuristic analyses of concatenated sequences for all seven loci using PAUP version 4.10b (Sinauer Associates, Sunderland, MA, USA), excluding uninformative characters, and employing ST 41 (*Bacillus weihenstephanensis*) as an outgroup to root the tree after alignment using CLUSTAL W algorithm of the MegAlign component of the Lasergene suite (DNASTAR, Inc. Madison, WI, USA).

Phylogenetic analysis of TcaC proteins among bacteria possessing *tca* operons was conducted to examine the phylogenetic inheritance of *tc* genes using using parsimony by branch and bound analyses using PAUP version 4.10b, excluding uninformative characters, and employing (*Burkholderia pseudomallei*) as the outgroup to root the tree after alignment. Bootstrap analyses (branch and bound) were then conducted to infer the character support for each clade.

### Optical mapping of plasmids

Optical mapping, a technique for generating restriction maps on a genomic scale [18], was performed on IBL 200 and IBL 122 by OpGen Inc. (Gaithersburg, MD, USA) using the restriction enzyme XbaI. Partially assembled genomic sequences of IBL 200 (GenBank accession number NZ_CM000758) were aligned with the optical map of IBL 200, and optical maps of the two isolates were compared, using MapSolver software (OpGen, Gaithersburg, MD, USA).

### Insect toxicity

Five *tc*-positive *Bt* isolates were selected to be evaluated for toxicity to gypsy moth, *Lymantria dispar* (L.), larvae. These included IBL 90, IBL 26, IBL 747, IBL 122, and IBL 3090. IBL 455, a lepidopteran-active strain isolated from a 1980 preparation of Dipel® (Abbot Laboratories, Chicago, IL, USA) was included as a standard for toxicity to gypsy moth. All isolates were grown in T3 medium [12] in baffled flasks at 24°C, and shaken at 200 rpm for 4 d.

Insects were obtained from a laboratory colony (Otis ANGB, MA, USA) and were reared to the second instar on artificial diet [19]. Whole cultures of *Bt* were diluted by a factor of 500 (based on preliminary tests of IBL 455). Diluted cultures were used to rehydrate freeze-dried pellets (ca. 60 mg) of gypsy moth diet at a rate of 300 µl per pellet [20]. Twelve pellets were treated with each culture, plus a negative control (water only). Treated pellets were held in cells (1.6 cm diameter×1.6 cm deep) of plastic bioassay trays (Bio-BA-128©, Bio-Serv, Frenchtown, NJ, USA). Two early second instar larvae were placed on each pellet, for a total of 24 larvae per isolate. Larvae were held at 27°C for 6 d. Mortality was then recorded. The experiment was replicated four times. Proportion mortality was calculated, normalized by arcsine √% transformation, and analyzed by analysis of variance (ANOVA). Means were separated by the least significant difference (LSD) test [21].

Adult Colorado potato beetles were fed and allowed to oviposit on potato foliage. The eggs were harvested and hatched on freshly made Colorado potato beetle diet [22] with neomycin. Bioassays were conducted on freeze-dried pellets without neomycin as described previously [20]. Briefly, 16 diet pellets were placed in wells (1.6 cm diameter 1.6 cm deep) of white plastic bioassay trays (C–D International, Ocean City, NJ) and rehydrated with 300 µl of water (controls) or suspensions containing dilutions of the *B. thuringiensis* strains (IBL 61, IBL 122, IBL 441, IBL 747, IBL 950, IBL 1140 and IBL 3090). One newly molted second-instar Colorado potato beetle larva was added to each pellet. Wells were sealed with film and holes made in the film with insect pins. Larvae were weighed at 72 h; mean weights were compared by ANOVA and separated by LSD [21].

### Real-time PCR assays

Whole RNA was extracted from infected and non-infected gypsy moth larvae using the mirVana Kit (Ambion, Austin, TX, USA) to the stage of total RNA isolation, following the manufacturer's protocol. cDNAs were prepared using RETROscript (Ambion, Austin, TX, USA). Specific TaqMan® Primer/Probe assays were developed and used at a concentration of 1× per well (see Table S3). cDNA template of 0.5 ul/well for the 16s endogenous controls were used. A total of 2 ul/well of template was added to the remaining sample wells along with Taqman Universal PCR master mix at a concentration of 1× and water to a volume of 25 ul/well. Assays were conducted in triplicate on an ABI 7500 Real Time instrument using the following conditions; 50° for 2 minutes, 95° for 10 minutes, and then 95° for 15 seconds and 60° for 1 minute repeated 40 times. A two-step protocol was conducted with reverse transcription with gene-specific primers, followed by real-time qPCR with designed TaqMan® major groove binder probes. Relative quantification was performed using the comparative Ct method (24) for *cry1* using 16S as an endogenous internal control and log2-transformed gene/16S expression ratios for analysis, or by raw quantitation for *tcaA* and *tcaB*. For each, control reactions were conducted without reverse transcription and without template.

## Supporting Information

Table S1 Isolates utilized in the current study.
(DOC)

Table S2 Sequence types included in the phylogenetic analysis.
(DOC)

Table S3 Custom TaqMan Assay Bt gene-specific primers and reporter probes.
(DOC)
