# Supplementary material for: The Occurrence of Photorhabdus-Like Toxin Complexes in Bacillus thuringiensis
Source: PLoS One. 2011 Mar 25;6(3):e18122. doi: 10.1371/journal.pone.0018122 (PMC3064592; doi:10.1371/journal.pone.0018122)
Supplement: Table S1 — Isolates utilized in the current study. (DOC) [file pone.0018122.s001.doc]

**Table S1. Isolates utilized in the current study.**

| **Isolate** | **Phenotype^1^** | ***tccC*** | **Country** |
| --- | --- | --- | --- |
| **IBL 4** | **Ø** | **-** | **Nevada, USA** |
| **IBL 1302** | **Ø** | **-** | **Turkey** |
| **IBL 1306** | **Ø** | **-** | **California, USA** |
| **IBL 3476** | **Ø** | **-** | **Maryland, USA** |
| **IBL 3477** | **L** | **-** | **Maryland, USA** |
| **IBL 631** | **LSAE** | **-** | **Morocco** |
| **IBL 674** | **LSAE** | **-** | **India** |
| **IBL 717** | **LSAE** | **-** | **USA** |
| **IBL 1211** | **LSAE** | **-** | **Maryland, USA** |
| **IBL 3268** | **LSAE** | **-** | **Nepal** |
| **IBL 77** | **T** | **-** | **Wyoming, USA** |
| **IBL 117** | **T** | **-** | **Wyoming, USA** |
| **IBL 1677** | **T** | **-** | **Wyoming, USA** |
| **IBL 3090** | **T** | **+** | **New York, USA** |
| **IBL 66** | **TL** | **-** | **Illinois, USA** |
| **IBL 429** | **TL** | **-** | **South Korea** |
| **IBL 431** | **TL** | **-** | **Spain** |
| **IBL 3241** | **TL** | **-** | **Norway** |
| **IBL 441** | **TLA** | **-** | **Argentina** |
| **IBL 966** | **TLA** | **-** | **Nepal** |
| **IBL 1252** | **TLA** | **-** | **South Korea** |
| **IBL 2408** | **TLA** | **-** | **Spain** |
| **IBL 3** | **TLAE** | **-** | **Nevada, USA** |
| **IBL 159** | **TLAE** | **-** | **New Mexico, USA** |
| **IBL 1247** | **TLAE** | **-** | **South Korea** |
| **IBL 1254** | **TLAE** | **-** | **South Korea** |
| **IBL 3565** | **TLAE** | **-** | **Maryland, USA** |
| **IBL 1268** | **TLE** | **-** | **Argentina** |
| **IBL 184** | **TLS** | **-** | **District of Columbia, USA** |
| **IBL 416** | **TLS** | **-** | **China** |
| **IBL 520** | **TLS** | **-** | **Florida, USA** |
| **IBL 1297** | **TLS** | **-** | **Maryland, USA** |
| **IBL 425** | **TLSA** | **-** | **New York, USA** |
| **IBL 480** | **TLSA** | **-** | **Sweden** |
| **IBL 533** | **TLSA** | **-** | **Vietnam** |
| **IBL 695** | **TLSA** | **-** | **Hawaii, USA** |
| **IBL 3576** | **TLSA** | **-** | **Pennsylvania, USA** |
| **IBL 535** | **TLSAE** | **-** | **Maryland, USA** |
| **IBL 1132** | **TLSAE** | **-** | **Norway** |
| **IBL 1489** | **TLSAE** | **-** | **Spain** |
| **IBL 1491** | **TLSAE** | **-** | **Spain** |
| **IBL 1613** | **TLSAE** | **-** | **Spain** |
| **IBL 673** | **TLSE** | **-** | **New Hampshire, USA** |
| **IBL 26** | **TLU** | **+** | **Wisconsin, USA** |
| **IBL 54** | **TLU** | **+** | **Montana, USA** |
| **IBL 58** | **TLU** | **-** | **Wyoming, USA** |
| **IBL 61** | **TLU** | **+** | **Montana, USA** |
| **IBL 110** | **TLU** | **+** | **New York, USA** |
| **IBL 200** | **TLU** | **+** | **Maryland, USA** |
| **IBL 357** | **TLU** | **-** | **Hawaii, USA** |
| **IBL 420** | **TLU** | **-** | **Jamaica** |
| **IBL 537** | **TLU** | **-** | **New Zealand** |
| **IBL 563** | **TLU** | **-** | **North Carolina, USA** |
| **IBL 635** | **TLU** | **-** | **Maryland, USA** |
| **IBL 888** | **TLU** | **+** | **Iceland** |
| **IBL 1140** | **TLU** | **+** | **Norway** |
| **IBL 1288** | **TLU** | **-** | **Azores** |
| **IBL 1380** | **TLU** | **-** | **Nepal** |
| **IBL 3087** | **TLU** | **-** | **Wyoming, USA** |
| **IBL 55** | **TLUA** | **-** | **Wyoming, USA** |
| **IBL 90** | **TLUA** | **+** | **Wyoming, USA** |
| **IBL 743** | **TLUA** | **-** | **Maryland, USA** |
| **IBL 999** | **TLUA** | **-** | **Maryland, USA** |
| **IBL 1115** | **TLUA** | **+** | **Sweden** |
| **IBL 3317** | **TLUA** | **-** | **Jamaica** |
| **IBL 84** | **TLUAE** | **-** | **Wisconsin, USA** |
| **IBL 88** | **TLUAE** | **-** | **North Dakota, USA** |
| **IBL 455** | **TLUAE** | **-** | **USA** |
| **IBL 661** | **TLUAE** | **-** | **Pennsylvania, USA** |
| **IBL 749** | **TLUAE** | **-** | **Nepal** |
| **IBL 1405** | **TLUE** | **-** | **Nepal** |
| **IBL 122** | **TS** | **+** | **Wyoming, USA** |
| **IBL 500** | **TS** | **+** | **Norway** |
| **IBL 747** | **TS** | **+** | **New York, USA** |
| **IBL 1410** | **TS** | **-** | **USA** |
| **IBL 3349** | **TS** | **-** | **Maryland, USA** |
| **NRRL-30759** | **TS** | **+** |  |
| **NRRL-30759** | **TS** | **+** |  |
| **IBL 273** | **TSAE** | **+** | **Wyoming, USA** |
| **IBL 275** | **TSAE** | **+** | **Wyoming, USA** |
| **IBL 3579** | **TSAE** | **+** | **New York, USA** |
| **IBL 950** | **TU** | **+** | **North Carolina, USA** |
| **NRRL-B-30758** | **TU** | **+** |  |
| **IBL 70** | **TUS** | **-** | **Hawaii, USA** |

^1^Letters correspond to the following traits: T-amylase production; L-lecithinase production; S-acid production from sucrose; A-acid production from salicin; E-hydrolysis of esculin; U-urease production; **Ø**-negative for all traits.
