# Supplementary material for: The Occurrence of Photorhabdus-Like Toxin Complexes in Bacillus thuringiensis
Source: PLoS One. 2011 Mar 25;6(3):e18122. doi: 10.1371/journal.pone.0018122 (PMC3064592; doi:10.1371/journal.pone.0018122)
Supplement: Table S2 — Sequence types included in the phylogenetic analysis. (DOC) [file pone.0018122.s002.doc]

Table S2. Sequence types included in the phylogenetic analysis.

| ST-1, ST-2, ST-3, ST-4, ST-8, ST-10, ST-12, ST-13, ST-15, ST-16, ST-18, ST-19,  ST-22, ST-23, ST-24, ST-25, ST-26, ST-32, ST-33, ST-41, ST-49, ST-55, ST-56,  ST-61, ST-65, ST-67, ST-69, ST-70, ST-72, ST-73, ST-78, ST-97, ST-109, ST-111,  ST-117, ST-142, ST-144, ST-160, ST-164, ST-167, ST-172, ST-177, ST-196, ST-205,  ST-223, ST-240, ST-268, ST-272, ST-274, ST-278, ST-281, ST-286, ST-295, ST-341,  ST-365, ST-370, ST-371, ST-374, ST-376, ST-387, ST-395, ST-471, ST-480, ST-481,  ST-503, ST-545 |
| --- |
