# Supplementary material for: The Occurrence of Photorhabdus-Like Toxin Complexes in Bacillus thuringiensis
Source: PLoS One. 2011 Mar 25;6(3):e18122. doi: 10.1371/journal.pone.0018122 (PMC3064592; doi:10.1371/journal.pone.0018122)
Supplement: Table S3 — Custom TaqMan Assay Bt gene-specific primers and reporter probes. (DOC) [file pone.0018122.s003.doc]

Table S3. Custom TaqMan Assay Bt gene-specific primers and reporter probes

| ASSAY NAME | FORWARD PRIMER SEQ. | REVERSE PRIMER SEQ | REPORTER 1 SEQUENCE |
| --- | --- | --- | --- |
| CRY1 | GTGTCAATGCGGCTATTTTTGAAGA | CACCATTTTTAATGACATTTCTCGCATCA | TTCACTGCATTCTCCC |
| TCAA | GCCGTCAAAAGGACAGAAAATTTAAGA | AGGATAATTTTGGAATATCTTTGAAAATTGGATCATTG | AAGGCATGGCAACTAC |
| TCAB | CCAGAAAAATGGACTCAGTGGGAAA | GCACATGCAAGCGTTTTTTATAACAGTAA | CTTCGCCGATATTCG |
| 16S-ASSAY | CTTTCTGGTCTGTAACTGACACTGA | GTGGACTACCAGGGTATCTAATCCT | CTCCCCACGCTTTCG |
